# Interferon Gamma Release Assay versus Tuberculin Skin Testing among Healthcare Workers of Highly Diverse Origin in a Moderate Tuberculosis Burden Country

**DOI:** 10.1371/journal.pone.0154803

**Published:** 2016-05-05

**Authors:** Sahal Al Hajoj, Bright Varghese, Alria Datijan, Mohammed Shoukri, Ali Alzahrani, Abdallah Alkhenizan, Abdulaziz AlSaif, Sahar Althawadi, Grace Fernandez, Abdulrahman Alrajhi

**Affiliations:** 1 Mycobacteriology Research Section, Department of Infection and Immunity, King Faisal Specialist Hospital and Research Centre, Riyadh, Saudi Arabia; 2 National Biotechnology Centre, King Faisal Specialist Hospital and Research Centre, Riyadh, Saudi Arabia; 3 Gulf Centre for Cancer Control and Prevention, King Faisal Specialist Hospital and Research Centre, Riyadh, Saudi Arabia; 4 Department of Family Medicine, King Faisal Specialist Hospital and Research Centre, Riyadh, Saudi Arabia; 5 Department of Pathology and Laboratory Medicine, King Faisal Specialist Hospital and Research Centre, Riyadh, Saudi Arabia; 6 Department of Medicine, King Faisal Specialist Hospital and Research Centre, Riyadh, Saudi Arabia; University of Hyderabad, INDIA

## Abstract

Health care workers (HCW’s) are always at an increased risk of contracting tuberculosis (TB) infection. In Saudi Arabia, Interferon Gamma Release Assay (IGRA) has not been evaluated as a screening tool for latent TB infection (LTBI) among HCW’s considering their high demographic diversity. During February 2012 to January 2015 a cross sectional study has been conducted in a tertiary care center with maximum demographically diverse staff population in the capital city-Riyadh. After a short interview and consenting, all the candidates were subjected to tuberculin skin test (TST) and QuantiFERON TB gold In-tube test (QFT). A logistic regression analysis was carried out for establishing the associations between putative risk factors and the diagnostic tests. The candidates were classified according to geographical origin and a detailed analysis was conducted on the impact of their origin towards the results of TST and QFT. Of the 1595 candidates enrolled, 90.6% were BCG vaccinated, female (67.9%) and mainly nurses (53.2%). Candidates with high risk of suspected or confirmed TB patient exposure were 56.1% and 76.5% of them had <10 year’s work experience. TST positivity was observed in 503 (31.5%) candidates, while QFT was positive among 399 (25%). Majority of the candidates were non-Saudi (83%) and predominantly (52.4%) from Western Pacific region. Concordant results were obtained in 14.2% of positive cases and 57.7% negative cases. The disagreements between the two tests were relatively high (kappa co-efficient-0.312±0.026, p value- <0.00001) as TST positive/QFT negative discordance was 54.8% while TST negative/QFT positive discordance was 15.7%. Age of the candidates, BCG vaccination, and South East Asian origin were associated with TST positivity while Occupational TB exposure and geographical origin of the candidates were associated with QFT positivity. A regular follow up on recently TST converted candidates showed no progression to active TB. The putative factors associated with the discordance were origin of the candidate (p value <0.001), profession (p value-0.001), BCG vaccination (p value-0.001) and occupational TB exposure level (P value-0.001). The study demonstrated high level prevalence of LTBI among the demographically diverse study candidates. The agreement between QFT and TST was poor, thus QFT alone cannot be recommended in our setting for a routine LTBI screening. Origin of the candidates has strong association with the results of TST and QFT. The discordant results particularly TST negative and QFT positive needs more detailed analysis.

## Introduction

Tuberculosis (TB) continues to be a major global health problem including in Saudi Arabia [1]. High proportion of immigrants living in the country and annual massive influx of pilgrims mostly from TB endemic areas constitute favorable conditions for TB transmission [2–4]. One of the most important challenges in global TB control is the early detection and treatment of latent tuberculosis infection (LTBI). About 10% of individuals infected with *M*. *tuberculosis* develop pulmonary TB, and the remaining 90% suppress the bacterial invasion through their immune systems and persist with LTBI [5]. Unfortunately there is very limited data on the prevalence of LTBI among health care workers in Saudi Arabia, except reports from few institutions [6].

Health care workers (HCW’s) in general are considered as a high-risk group of LTBI because of the increased risk of exposure [7–9]. A recent systematic review showed, among HCW’s of low and middle income countries, the LTBI prevalence is ranged between 33–79% with a median of 5.8% annual LTBI incidence [10]. Usually, HCW’s may come into contact with patients with undiagnosed cases or unknown/unsuspected cases of active TB, which elevates the acquisition of LTBI. HCW’s employed in sections like emergency, intensive care, internal medicine, radiology, are at greater risk of acquiring *M*. *tuberculosis*. Due to the epidemiological evidence of TB as a consequential occupational disease, HCW’s with negative TST results must undergo annual LTBI screening.

Surveillance study on LTBI among HCW’s vaccinated with BCG has been hampered by the non- specificity of TST. False-positive results secondary to cross-reactions caused by BCG vaccination and/or exposure to non tuberculous mycobacteria, booster phenomenon and technical issues, like interpretation of the result may all lead to unnecessary treatment of presumed LTBI thereby, rendering TST unsuitable as a surveillance tool in TB risk groups [11,12]. The advent of Interferon Gamma Release Assays (IGRA) and their increasing availability show promise for more accurate LTBI detection in HCW’s. The higher specificity of QuantiFERON TB gold assay (QFT) compared with TST can reduce unnecessary treatment, follow-ups and thereby, treatment cost of LTBI [13,14].

Most IGRA studies have been done in low TB-endemic regions whereas; data from low to middle-income settings, with high background of TB infection rates have been fairly scarce. We are not aware of any report describing the use of IGRA’s among HCWs in Saudi Arabia or in Gulf Cooperation Council (GCC) countries.

In Saudi Arabia, TB screening is recommended for all HCW’s. Annual LTBI screening is mandatory for all employees at King Faisal Specialist Hospital and Research Centre (KFSHRC) as part of their job contract renewals. The TST is the currently following LTBI diagnostic method at KFSHRC. However due to the limitations of the TST and to promote a more sensitive technique, IGRA was considered. The cosmopolitan nature of the employee population (citizens of more than 60 countries) has not been considered for optimizing the LTBI diagnosis. Moreover, the majority of the employee populations are from countries where BCG vaccination is mandatory. In addition, there was an increasing prevalence of NTM diseases also reported in the country recently [15]. Thus a new LTBI diagnostic test was badly needed for the institution.

Therefore, a cross sectional study has been designed with two major set objectives. The key objectives were to analyze the prevalence of LTBI among HCW’s of highly diverse origin at KFSHRC and to compare the feasibility of using TST and QFT to screen the LTBI among this diverse population.

## Materials and Methods

This study was carried out for a period of 36 month (February 2012 to January 2015) in KFSHRC, Riyadh. The protocol has been reviewed and approved by the Research Advisory Council (RAC) of KFSHRC. All the candidates enrolled were subjected to a short interview and completed the consent form before withdrawing the blood samples.

### Study population

The inclusion criteria for study subjects was any new (undergoing pre-employment checkup) or existing employee (annual re-contracting checkup), who can consent for the study. The exclusion criteria included candidates with previous history of active TB or undergoing TB medication. The study subjects were recruited from both clinical and non-clinical health care workers designated in various departments at KFSHRC. Clinical health-care workers (those included in any direct patient contact or giving direct care) are doctors, nurses and allied health professionals. Non-clinical health-care workers included those who did not involve in direct patient care, such as administrative staffs, researchers, housekeepers, and hospital technical maintenance staffs.

According to the job profile of the candidate, the degree of TB exposure level was defined into low, medium and high. Doctors, nurses and allied health professionals were classified into high risk group. The medium risk group consisted of candidates who are not directly giving patient care but in contact with patient samples (ex; medical lab technicians) or technicians managing respiratory units or equipments. The low risk group included researchers, maintenance staffs and administrative staffs respectively.

A standard questionnaire was used to collect the information on key variables, such as age, gender, nationality, previous exposure or treatment details of TB, previous chest x-ray details, BCG vaccination and BCG scar, prior TST (date and result), job category, service and years in the health profession. The questionnaire has been completed by face to face interview and reference to medical records.

All the TST positive converted staffs were offered with a follow up of Chest X-ray and a 9 months isoniazid treatment according to the standard guidelines. The decision of treatment was kept optional to the employee after the discussion with the physician. However a continuous follow-up was mandatory.

### Performing TST and QFT

All new HCW’s, who did not have a documented TST result, were subjected to TST during the routine examination at the time of employment in the family medicine department of KFSHRC. TST was performed by administrating 2-TU of PPD RT23 (Staten’s Serum Institute, Copenhagen, Denmark). Induration was measured 48–72 h after the application. Well trained nurses performed and interpreted the results in 48–72 hours according to the American Thoracic Society (ATS) and Centers for Disease Control and Prevention guidelines [16]. The positive interpretation followed was a TST with an area of induration ≥10 mm. If HCW’s had a previous positive TST, we took note of the place and the year. If HCW’s had negative TST, it is repeated annually or after an exposure as part of KFSHRC’s infection control practice.

The QFT was carried out by using the commercially available QuantiFERON TB gold In-Tube Assay (Cellestis, Australia) according to the manufacturer’s protocol. The raw optical density was measured and interpreted with the software QuantiFERON TB Gold analysis Software v1.51 (Cellestis, Australia).

#### Statistical analysis

The candidates were classified according to the WHO’s geographical classification into 6 major groups; American, African, European, Eastern Mediterranean, South East Asian, and Western Pacific [17]. The statistical analysis has been carried out by using the SPSS v-20 software package. A chi squared test was utilized for categorical data. All the putative predictive variables were subjected to calculate the odds ratio and 95% confidence interval.

## Results

Overall 1603 candidates were enrolled during the study period. Of the total, 8 HCWs (1.2%) were excluded from the analysis due to indeterminate QFT results and 1595 were included for final analysis. The study population included candidates from 33 countries. The native Saudi population was only 17%. The geographical classification on enrolled candidates showed, 52.4% were from Western-Pacific followed by 22.9% Eastern Mediterranean and 11.3% South East Asians. The median of age in the study was 35.5 years with a predominance of female candidates (67.9%). Clinical staffs represented the major study group by71.5% and among them nurses were predominant (53.2%). Overall, 185(11.6%) candidates reported with a previous exposure to known pulmonary TB cases. Majority of the candidates (90.6%) received at least one shot of BCG vaccination before enrollment (S1 Table). Candidates holding a healthcare experience of 2–5 years were comparatively more (37.6%), though the mean duration of experience in the study was 8.4 years (Table 1).

**Table 1:** General Characteristics of the study population.

Parameters	No/%
**Gender**	
**Male**	512(32.1)
**Female**	1083(67.9)
Age Group	
**19–29**	543 (34)
**29–39**	596(37.4)
**39–49**	292(18.3)
**49–59**	129(8.1)
**>60**	35(2.2)
Profession	
**Physician**	44(2.8)
**Nurses**	849(53.2)
**Allied Health**	248(15.5)
**Support Services**	348(21.8)
**Administration**	62(3.9)
**Researchers**	44(2.8)
Occupational degree of TB exposure	
**Low**	398(25)
**Medium**	302(18.9)
**High**	895(56.1)
Known Pulmonary TB exposure	
**Yes**	185(11.6)
**No**	556(34.9)
**Unknown**	854(83.5)
TST Converters	
**Yes**	170
**No**	1425
Prophylaxis among converted candidates	
**Yes**	46(2.9)
**No**	1545(96.9)
TST Result	
**Positive**	502(31.5)
**Negative**	1093(68.5)
QFT Results	
**Positive**	399(25)
**Negative**	1196(75)
BCG Vaccination	
**Yes**	1444(90.6)
**No**	92(5.8)
**Unknown**	58(3.6)
TST Induration	
**<10mm**	1093(68.5)
**10-15mm**	328(20.6)
**16-20mm**	116(7.3)
**>20mm**	58(3.6)
Years of work	
**<2 years**	108(6.8)
**2–5 years**	600(37.6)
**5–10 years**	511(32.1)
**10–20 years**	289(18.1)
**>20 years**	87(5.4)

**Fig 1:**
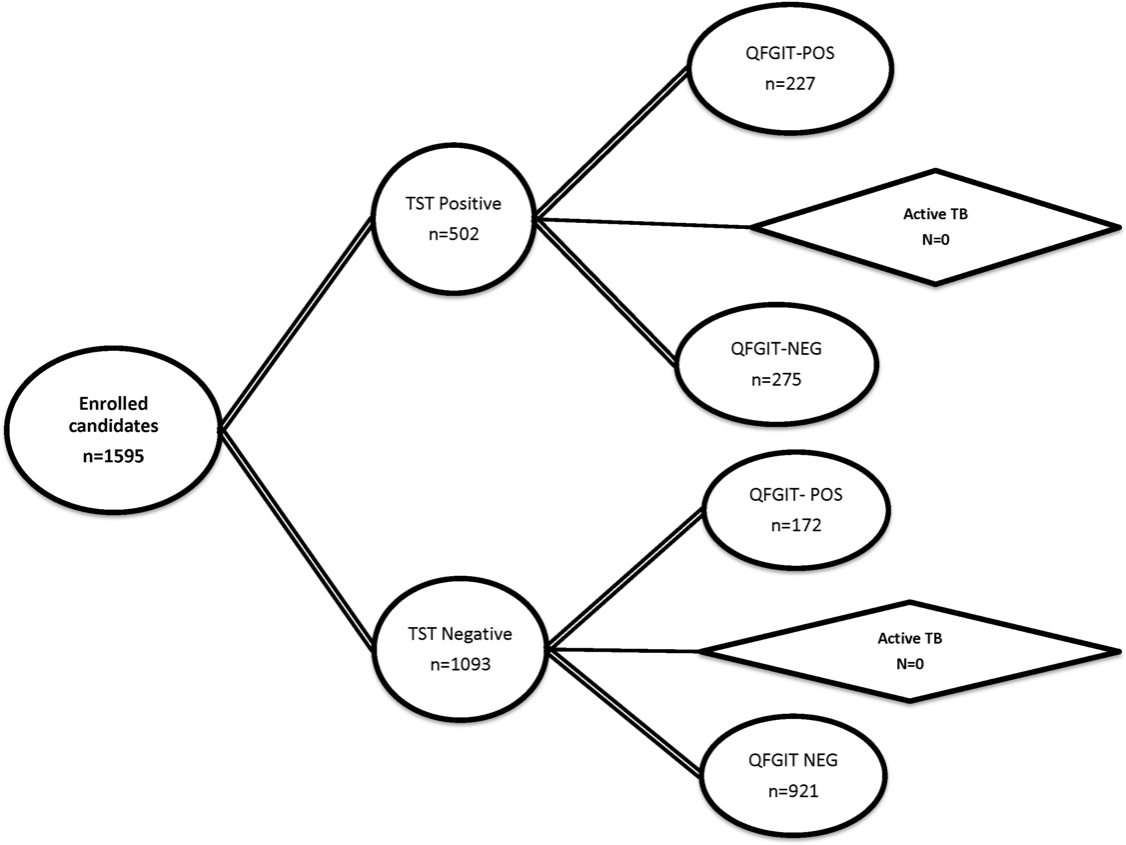
Study summary diagram with results. The figure shows the total number of samples tested (omitted 8 isolates because of Indeterminate QFT results) and the varying results of IGRA and TST. Progression of the candidates to active TB was also followed up with the concordance of test results.

Positive TST was noticed in 31.5% of the enrolled candidates. Among the TST positive cases 45.2% were QFT positive while 54.8% were negative. On the other hand, among TST negative cases, 15.7% were QFT positive. The disagreements between the two tests were relatively high (kappa co-efficient-0.312±0.026, p value- <0.00001). Among 170 TST converted participants only 46 opted for INH prophylaxis and none of these cases progressed to active TB during the study period (Fig 1). The regression analysis of the putative predictable factors showed a significant association of younger age groups (*p* -0.030, 0.049), BCG vaccination (*p*- 0.028), European (*p* -<0.001) and south East Asian (*p*-0.009) origin with TST positivity. QFT positivity was associated with profession of candidates and occupational TB exposure risk. In addition, the origin of the candidates (*p*-<0.001) was highly significant in QFT positivity (Table 2).

**Table 2:** Risk factors associated with positive TST and QFT.

Parameters	TST >10mm				QFT-Positive			
	N	OR	95%CI	P value	N	OR	95%CI	P value
Gender		1.021	0.814–1.279	0.859		0.952	0.747–1.212	0.688
**Male**	349				132			
**Female**	743				267			
Age group								
**19–29**	176	2.902	1.107–7.606	0.030	141	1.172	0.521–2.641	0.701
**29–39**	196	2.940	1.123–7.694	0.028	149	1.125	0.500–2.530	0.776
**39–49**	90	2.673	1.005–7.114	0.049	69	1.044	0.454–2.404	0.919
**49–59**	35	2.234	0.803–6.215	0.124	32	1.113	0.460–2.696	0.812
**>60**	6	REF			8	REF		
Profession								
**Physician**	11	0.583	0.233–1.461	0.250	9	0.613	0.231–1.630	0.327
**Nurses**	260	0.772	0.411–1.452	0.423	204	0.754	0.387–1.469	0.754
**Allied Health**	79	0.818	0.419–1.598	0.557	42	0.486	0.235–1.007	0.052
**Support Services**	107	0.777	0.404–1.496	0.450	111	1.117	0.563–2.217	0.752
**Administration**	30	1.641	0.744–3.618	0.220	20	1.054	0.453–2.448	0.903
**Researchers**	16	REF			13	REF		
Occupational Risk								
**Low**	136	REF			127	REF		
**Medium**	96	0.898	0.653–1.235	0.507	58	0.507	0.355–0.724	<0.001
**High**	271	0.837	0.651–1.076	0.165	214	0.666	0.513–0.865	0.002
Years of work								
**<2 Years**	33	1.306	0.655–2.602	0.448	26	1.306	0.655–2.602	0.448
**2–5 years**	194	1.536	0.878–2.688	0.133	163	1.536	0.878–2.688	0.133
**6–10 years**	164	1.264	0.716–2.230	0.419	120	1.264	0.716–2.230	0.419
**10–20 years**	95	1.366	0.755–2.473	0.303	72	1.366	0.755–2.473	0.303
**>20 years**	17	REF			17	REF		
BCG Vaccination	449	1.039	0.664–1.625	0.028	356	1.364	0.866–2.146	0.179
Geographical Origin								
**American**	28	1.236	0.760–2.011	0.394	13	0.481	0.260–0.889	0.019
**African**	18	0.671	0.388–1.162	0.154	12	0.444	0.235–0.837	0.012
**European**	37	3.201	1.888–5.427	<0.001	32	2.525	1.501–4.246	<0.001
**Eastern Mediterranean**	117	1.020	0.784–1.328	0.881	60	0.466	0.340–0.637	<0.001
**South East Asian**	39	0.598	0.408–0.878	0.009	33	0.531	0.354–0.797	0.002
**Western Pacific**	264	REF			249	REF		

Analysis of agreement of results of TST and QFT showed 14.23% concordant positive results while 57.7% is concordant negative. However, 10.8% cases were TST negative and QFT positive. On the other hand 275(17.2%) cases were TST positive and QFT negative. Analyzing the association of various putative factors showed the origin of the patient (*p* <0.001), occupational TB exposure level (*p*-<0.001), profession (*p-* 0.001) and BCG vaccination history (*p-* 0.001) have a statistical significance on the agreement of both the tests (Table 3). Among the discordant results, candidates with Western Pacific origin showed a predominant QFT positivity (65.7%) and negativity (46.9%). Interestingly, among the different professions, nurses have the highly discordant results (Table 3).

**Table 3:** Relationship between risk factors and pattern of concordant and discordant test results.

Parameters	N	Concordant Positives	Concordant Negatives	Discordant; TST-/QFT+	Discordant; QFT-/TST+	P value
		N = 227	N = 921	N = 172	N = 275	
Gender						0.980
**Male**	512	74(32.6)	292(31.7)	57(33.1)	89(32.4)	
**Female**	1083	153(67.4)	629(68.3)	115(66.9)	186(67.6)	
Age groups						
**19–29**	543	85(37.5)	311(33.8)	56(32.6)	91(33.1)	0.463
**29–39**	596	80(35.2)	331(35.9)	69(40.1)	116(42.2)	
**39–49**	292	42(18.5)	175(19)	27(15.7)	48(17.4)	
**49–59**	129	18(7.9)	80(8.7)	14(8.1)	17(6.2)	
**>60**	35	2(0.9)	24(2.6)	6(3.5)	3(1.1)	
Origin						<0.001
**American**	77	7(3.1)	43(4.7)	6(3.5)	21(7.6)	
**African**	76	7(3.1)	53(5.7)	5(2.9)	11(4.0)	
**European**	62	25(11.0)	18(1.9)	7(4.1)	12(4.4)	
**Eastern Mediterranean**	365	31(13.7)	219(23.8)	30(17.4)	85(30.9)	
**South East Asian**	180	22(9.6)	130(14.1)	11(6.4)	17(6.2)	
**Western Pacific**	835	135(59.5)	458(49.7)	113(65.7)	129(46.9)	
Profession						0.001
**Physician**	44	4(1.8)	28(3.0)	5(2.9)	7(2.5)	
**Nurses**	849	126(55.5)	511(55.5)	78(45.3)	134(48.7)	
**Allied Health**	248	25(11)	152(16.6)	18(10.6)	53(19.3)	
**Support Services**	348	54(23.8)	184(19.9)	57(33.1)	53(19.3)	
**Administration**	62	11(4.8)	24(2.6)	8(4.6)	19(6.9)	
**Researchers**	44	7(3.1)	22(2.4)	6(3.5)	9(3.3)	
Occupational Risk						<0.001
**Low**	398	64(28.2)	199(21.6)	64(37.2)	71(25.8)	
**Medium**	302	33(14.5)	181(19.6)	25(14.5)	63(22.9)	
**High**	895	130(57.3)	541(58.8)	83(48.3)	141(51.3)	
Years of work						0.153
**<2 Years**	108	12(5.3)	61(6.6)	15(8.7)	20(7.3)	
**2–5 years**	600	99(43.6)	342(37.2)	64(37.2)	95(34.6)	
**6–10 years**	511	62(27.3)	289(31.4)	58(33.7)	102(37.1)	
**10–20 years**	289	47(20.7)	169(18.3)	25(14.5)	48(17.4)	
**>20 years**	87	7(3.1)	60(6.5)	10(5.8)	10(3.6)	
BCG Vaccination	1444	206(90.7)	845(91.7)	151(87.8)	206(74.9)	0.001

## Discussion

This is the largest Saudi Arabian study, which evaluated the performance of QFT and TST among highly diverse HCW’s population. The study has targeted onto the putative risk factors with an emphasis on the impact of geographic origin of study subjects. It is also the only cross sectional study in the country that looked at the possibilities of developing active TB in recently identified LTBI in HCW’s. This study has been carried out in a tertiary care center which has been listed among the top five medical facilities in the country employing staffs from more than 60 countries. The impact of this huge diversity in the origin of the staffs has been highly considered on the LTBI prevalence, as it influence the agreement between TST and IGRA. The overall agreement between TST and QFT was 72%, that can be considered as fair, when many previous international studies reported moderate or fair agreement only between both the tests [18–20]. Supportively, recent studies from Saudi Arabia on dialysis patients also showed 75.5% and 90.9% agreement between TST and QFT/TSPOT Assay respectively [21,22].

The prevalence of LTBI measured in this study by TST (31.5%) and QFT (25.0%) was relatively high. This elevated rate may be typical for a moderate TB burden country like Saudi Arabia. Furthermore, the previous estimates showed that, Saudi Arabia has only an intermediate prevalence of LTBI (2–14%) [23]. In Saudi Arabia, only limited information is available on prevalence of LTBI among HCW’s particularly screened with IGRA testing. A multicenter study utilizing TST alone in HCW’s showed 11% LTBI prevalence, while the current study showed 31.5% [6]. However, the elevated prevalence level of LTBI in the current study may largely depend on the origin of the candidates, that particularly from TB endemic regions.

The current study population consisted only 17% Saudi nationals. The results of TST and QFT stratified by the Saudi and non-Saudi origin showed a prevalence of 18.1% and 17.3% among Saudis. This finding highlights the massive role of immigrant HCW’s in the institution and mostly their origin from TB endemic countries. After applying the geographical region classification of WHO into the study, showed the Western Pacific origin candidates (mainly Philippines) as the largest group in the study followed by the Eastern Mediterranean and south East Asians. Interestingly, there are 970(60.8%) candidates truly from high TB burden countries namely, Philippines, India, Bangladesh, Pakistan, Sudan, South Africa, Eritrea, Ethiopia and Kenya. Analysis of the TST results against the nationality of the candidates, showed 305(60.6%) of the total 503 TST positive and 234 (58.6%) of the total 399 QFT positive candidates are from this group. The agreement between two results was really narrow (only 2%). Among the candidates from high TB burden countries only 94(9.7%) had TST conversion during their employment in the study center. This speculates the scope of a remote infection among those remaining 876(90.3%) candidates from their previous destination that is mostly their mother country. This finding has a support from molecular studies by Varghese et al., which showed remote TB infection among immigrants is very common Saudi Arabia [24,25].

In the current study the maximum discordance was observed in TST positive and QFT negative results. Among the TST positive cases 54.7% were negative for QFT. This finding corroborates with published studies which reported positive TST and negative IGRA is the most common discordance [26]. Majority of the study candidates were from a country where BCG vaccination is mandatory, thus 90.6% of the enrolled candidates were BCG vaccinated. This could probably affect the higher level prevalence of TST positivity. Supportively, a statistically significant association (OR 1.039, 95% CI-0.664–1.625, P value 0.028) was noticed between BCG vaccination and TST positivity while no significant association with QFT. This finding is in concordance with previous studies, which showed BCG vaccination significantly elevates the likelihood of TST positivity [27]. When compared with age >60 years, the younger age has significant association with TST positivity in concordance with various previous studies [28,29]. The rate of TST positivity among different professionals showed no significant association. In contrast, the TB exposure level based on profession showed a high significance towards QFT positivity, when low risk group was kept as a reference as seen in previous studies [30,31]. The origin of the candidates also has significant association with both TST and QFT positivity.

The maximum discordant results were noticed among candidates from Western Pacific region. Overall 65.7% of the TST negative QFT positive candidates and 46.9% of TST positive and QFT negative candidates were from mainly Philippines and Malaysia, while candidates from Eastern Mediterranean region showed the rates of 17.4% and 30.9% respectively. The massive enrollment of Filipino candidates (45.2%) has an impact on the overall findings. The two main reasons are the mandatory BCG vaccine administration and high TB burden of Philippines. Perhaps, the higher discordant results of TST negative and QFT positive could not be explained in detail. No significant association could be established between the discordant results against age of the candidates, gender, and years of work in the medical field. However, strong associations could be noticed on origin of the candidates (*p* <0.001), profession (*p* 0.001), TB exposure level (*p* <0.001) and BCG vaccination (*p* 0.001). Nurses and support service staffs showed majority of the discordant results. This finding was supported by the rate of TB exposure level as the high TB exposure group has highest discordance between results. This study has certain limitations; candidates reported as TST negative and QFT positive were not retested for confirmation and quantitative analysis of QFT testing was not considered.

## Conclusion

In conclusion, the prevalence of LTBI estimated by QFT is high among Saudi Arabian HCW’s. The disagreements between TST and IGRA results were relatively high and thus QFT alone cannot be recommended to screen LTBI. The origin of the candidates has significant role in both TST and IGRA positivity. However due to the high level of LTBI prevalence, the screening and management of LTBI in HCW’s in the country must be immediately streamlined.

## Supporting Information

S1 Table Data. (XLSX)
